# Comments on “Comparison of different neoadjuvant treatments for resectable locoregional esophageal cancer: A systematic review and network meta‐analysis”

**DOI:** 10.1111/1759-7714.14698

**Published:** 2022-10-18

**Authors:** Yuk‐Ming Tsang, Chen‐Xiong Hsu, Pei‐Wei Shueng

**Affiliations:** ^1^ Division of Medical Imaging, Department of Radiology Far Eastern Memorial Hospital New Taipei City Taiwan; ^2^ Division of Radiation Oncology, Department of Radiology Far Eastern Memorial Hospital New Taipei City Taiwan; ^3^ School of Medicine, College of Medicine National Yang Ming Chiao Tung University Taipei Taiwan

To the Editor

We read with great interest a current systematic review and network meta‐analysis by Bao and colleagues.^1^ The authors reported that neoadjuvant chemoradiation therapy followed by surgery (NCRT + S) could be the most efficient neoadjuvant therapy for resectable locoregional esophageal cancer by using the Bayesian network meta‐analysis. Previous randomized trials including the CROSS, CALGB9781, and FFCD 9901 have focused on the survival benefits of NCRT+S versus surgery alone (S); however, the results are variable. The CROSS and CALGB9781 trials demonstrated a significant survival benefit for NCRT+S group versus S,^2^, ^3^ whereas the FFCD 9901 trial reported that NCRT+S did not improve OS.^4^

We appreciated the analyses of Bao and colleagues,^1^ and the authors also found that NCRT + S could increase the risk of postoperative mortality but not postoperative morbidity. Nevertheless, some researchers also indicated that nonresponders to neoadjuvant chemoradiation therapy (NCRT) may experience no benefit and even worse outcomes than those undergoing primary S for locally advanced esophageal cancer. In addition, NCRT may lead to increased morbidity, toxicity, and a delay in curative surgery.^5^, ^6^ Finally, we thank the authors for their excellent work and also look forward to more clinical trials to investigate prognostic markers and precisely select the patients who will benefit from NCRT+S.

## CONFLICT OF INTEREST

The authors declare no conflict of interest.
